# Blocking Autophagy in Cancer-Associated Fibroblasts Supports Chemotherapy of Pancreatic Cancer Cells

**DOI:** 10.3389/fonc.2018.00590

**Published:** 2018-12-05

**Authors:** Xianbin Zhang, Maria Schönrogge, Johanna Eichberg, Edgar Heinz Uwe Wendt, Simone Kumstel, Jan Stenzel, Tobias Lindner, Robert Jaster, Bernd Joachim Krause, Brigitte Vollmar, Dietmar Zechner

**Affiliations:** ^1^Institute for Experimental Surgery, Rostock University Medical Center, Rostock, Germany; ^2^Core Facility Multimodal Small Animal Imaging, Rostock University Medical Center, Rostock, Germany; ^3^Division of Gastroenterology, Department of Medicine II, Rostock University Medical Center, Rostock, Germany; ^4^Department of Nuclear Medicine, Rostock University Medical Center, Rostock, Germany

**Keywords:** autophagy, cancer-associated fibroblasts, gemcitabine, metformin, α-cyano-4-hydroxycinnamate, pancreatic cancer

## Abstract

In this study we evaluated the interaction of pancreatic cancer cells, cancer-associated fibroblasts, and distinct drugs such as α-cyano-4-hydroxycinnamate, metformin, and gemcitabine. We observed that α-cyano-4-hydroxycinnamate as monotherapy or in combination with metformin could significantly induce collagen I deposition within the stromal reaction. Subsequently, we demonstrated that cancer-associated fibroblasts impaired the anti-proliferation efficacy of α-cyano-4-hydroxycinnamate, metformin and gemcitabine. Interestingly, inhibition of autophagy in these fibroblasts can augment the anti-proliferation effect of these chemotherapeutics *in vitro* and can reduce the tumor weight in a syngeneic pancreatic cancer model. These results suggest that inhibiting autophagy in cancer-associated fibroblasts may contribute to strategies targeting cancer.

## Introduction

Despite decades of intensive effort, the 5-years relative survival rate of pancreatic cancer is still only 8% (1). Although several treatment strategies showed promising preclinical anti-cancer activity, most of them failed to show significant efficacy in clinical trials. One reason might be that these treatment strategies only targeted pancreatic cancer cells, but ignored the abundant desmoplastic stroma around the tumor (2). This stromal reaction impairs vasculature and functions as a barrier to chemotherapeutics. Unfortunately, most preclinical pancreatic cancer models fail to replicate the dense stroma accurately (2).

An important cell type of the stromal reaction is the cancer-associated fibroblast (CAF). Several studies demonstrated that these fibroblasts contribute to tumor progression and chemoresistance in pancreatic cancer (3, 4). Thus, research has lately focused on the evaluation of drugs, which deplete CAFs (5–7). However, subsequent clinical trials demonstrated that reducing fibrosis in addition to a first-line therapy was not beneficial for patients (8). This suggests that reduction of CAFs is not a good option for cancer therapy. A better option may be to modify specific aspects of interactions between CAFs and carcinoma cells.

Lactate is an important mediator for the interaction of CAFs and cancer cells. For example, CAFs are stimulated by cancer cells to produce and export lactate. Subsequently, cancer cells import this lactate to fuel the Krebs cycle and support anabolic processes as well as cell proliferation (9). This interaction between CAFs and carcinoma cells can be blocked by α-cyano-4-hydroxycinnamate (CHC), an inhibitor of lactate transporter (9).

In addition, another process, called autophagy (10–12), is also involved in the interaction between tumor and CAFs (13–15). Recent evidence proved that CAFs promote tumor growth through autophagy, which is responsible for providing nutrients to carcinoma cells (13). Moreover, autophagy is often necessary for the survival of cells, especially when cells are starved or treated by chemotherapeutics (10, 11). Multiple studies have explored this effect of autophagy in pancreatic cancer (10, 12). However, it is unknown, if and how distinct chemotherapeutics influence autophagy in CAFs, or whether blocking the autophagic flux in CAFs can improve the efficacy of chemotherapies in pancreatic cancer cells.

It was the aim of this study to evaluate the interaction among drugs, carcinoma cells, and autophagy in CAFs. Specifically, we wanted to address the question if inhibiting autophagy in CAFs could support the anti-proliferation activity of metabolic inhibitors, such as metformin and CHC or gemcitabine, a first-line therapy for the treatment of pancreatic cancer.

## Materials and Methods

### Reagents and Antibodies

Dimethyl sulfoxide (DMSO, code D2438), CHC (code 476870), metformin (code D150959), gemcitabine (code G6423), mitomycin C (MCC, code M7949), and chloroquine (CQ, code PHR1258) were purchased from Sigma-Aldrich (St. Louis, USA). Bafilomycin A1 (BAF, code 196000) was obtained from Merck Millipore (Darmstadt, Germany). Primary antibodies against type I collagen (collagen I, code ab34710), p62 (code ab109012-100), and β-actin (code A5441) were obtained from Abcam (Cambridge, UK) or Sigma-Aldrich. Secondary antibodies, goat anti-rabbit immunoglobulins (code D0487), peroxidase linked anti-rabbit antibody (code 7074), peroxidase linked anti-mouse antibody (code A9044), and liquid permanent red system (code K0640) were purchased from Dako (Hamburg, Germany), Cell Signaling (Danvers, USA) or Sigma-Aldrich.

### Cell Culture and Treatment of Distinct Chemotherapeutic Strategies

The murine pancreatic adenocarcinoma cell line 6606PDA was a gift from Prof. Tuveson at the University of Cambridge, UK. The human pancreatic cancer cell line MIA PaCa-2 was purchased from ATCC (Manassas, USA). These cells were cultured in Dulbecco's Modified Eagle's Medium (DMEM, code FG0435, Biochrom, GmbH, Berlin, Germany) supplemented with 10% fetal calf plasma (FCS), 100 units/ml penicillin and 100 μg/ml streptomycin. The generation and characterization of immortalized rat pancreatic stellate cell line, LTC-Tet (LTC), were described previously (16, 17). These cells were cultured in Iscove's Modified Dulbecco's Medium (IMDM, code FG4605, Biochrom, GmbH, Berlin, Germany) supplemented with 10% FCS, 100 units/ml penicillin, 100 μg/ml streptomycin, and 1% non-essential amino acids (Sigma-Aldrich, code M7145). Cells were treated with 10 mM CHC, 5 mM metformin, both drugs, 0.1 μM gemcitabine or an appropriate vehicle (Sham) as indicated in each figure.

### Evaluation of Proliferation and Cell Death

In order to evaluate the benefit of CHC and metformin for treating pancreatic cancer, 2 × 10^3^ 6606PDA cells per well were seeded in a 96 well microplate. After 24 h, these cells were treated with the indicated chemotherapeutic agents for 48 h. To evaluate the function of CAFs *in vitro*, 1 × 10^4^ LTC cells per well were cultured in a 96 well microplate for 24 h. To stop proliferation, all LTC cells were treated with 5 μg/ml MMC for 3 h (see **Figures 3–5**). As indicated in **Figures 4**, **5C,D**, autophagy in LTC cells was inhibited by additionally pretreating with 50 μM CQ or 0.2 μM BAF for 3 h. These cells were then washed two times with phosphate buffered saline (PBS), and co-cultured with 2 × 10^3^ 6606PDA or 4 × 10^3^ MIA PaCa-2 cells per well for 24 h. Afterwards, these cells were treated with CHC, metformin, both drugs, or appropriate vehicle (Sham) for 48 h. Alternatively, these co-cultured cells were treated with gemcitabine or vehicle (Sham) for 24 h. Subsequently, the proliferation of 6606PDA cells was quantified by incorporation of 5-bromo-2'-deoxyuridine (BrdU) with colorimetric Cell Proliferation ELISA kit (Roche Diagnostics, Mannheim, Germany, code 11647229001) and Perkin Elmer Victor X3 model 2030 Multilabel Plate Reader platform (PerkinElmer, Waltham, USA).

To assess the synergistic effect of CHC and metformin in cell death, 3 × 10^4^ 6606PDA cells per well were plated in a 24 well plate. On the following day these cells were treated for 56 h with chemotherapeutic agents as indicated in **Figure 2B**. Subsequently, the percentage of dead cells was determined with the help of a trypan blue solution.

### Western Blot

For western blots, 2.4 × 10^5^ LTC cells per well were plated in a 6 well plate. After 24 h these cells were treated with distinct drugs as indicated in **Figure 7**; and then the western blots were performed as previously described using rabbit anti-p62 antibody (dilution: 8,000 × ), mouse anti-β-actin antibody (dilution: 20,000 × ), peroxidase linked anti-rabbit antibody (dilution: 10,000 ×) and peroxidase linked anti-mouse antibody (dilution: 60,000 ×). Proteins were visualized by luminol-enhanced chemiluminescence (ECL plus; GE Healthcare, Munich, Germany) and Chemi-Doc XRS System (Bio-Rad Laboratories, Munich, Germany) (18).

### Animals and the Syngeneic Orthotopic Pancreatic Cancer Model

**Figure 1:**
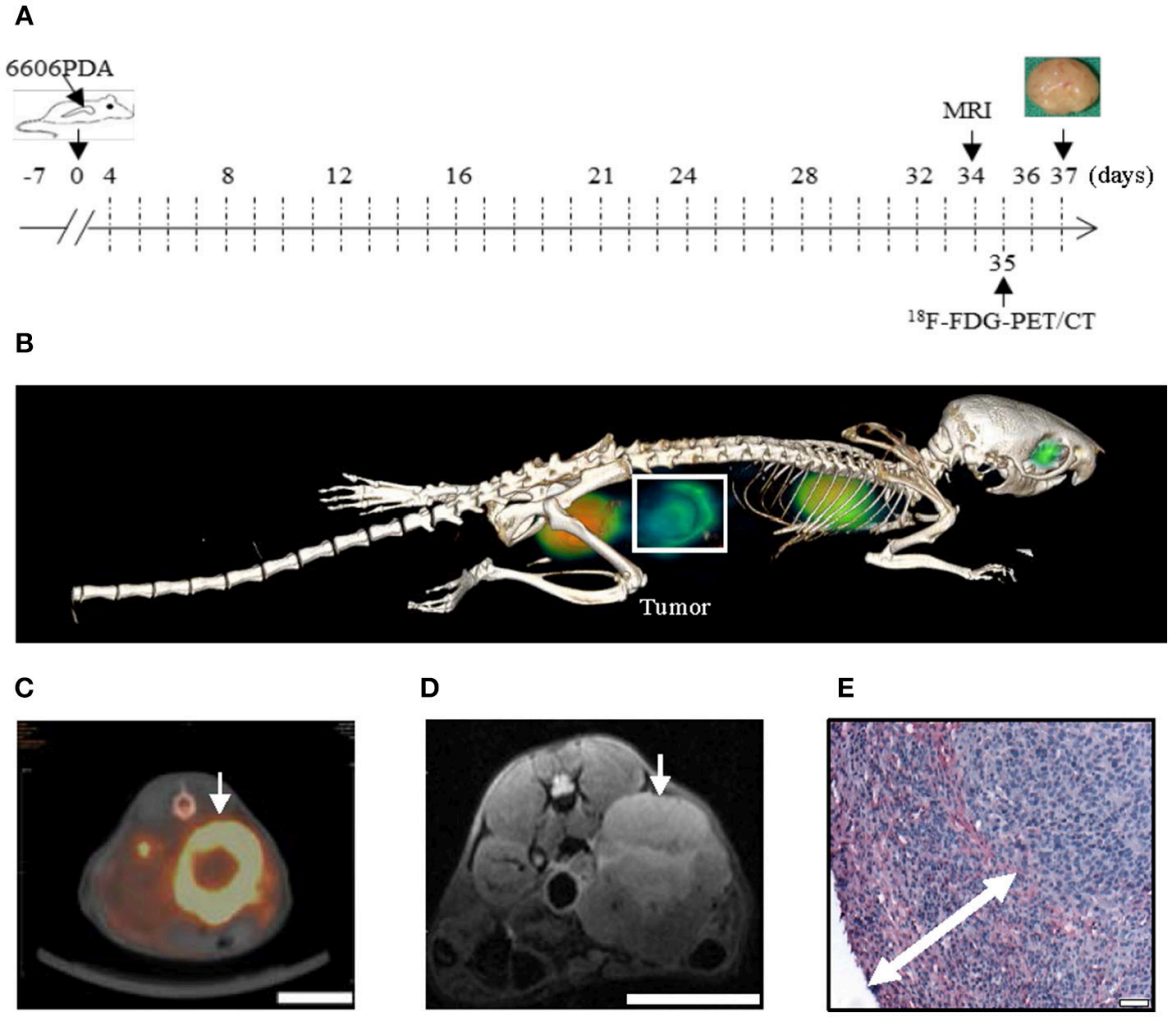
The 6606PDA syngeneic orthotopic pancreatic cancer model replicates features of human pancreatic cancer. 6606PDA cells were injected into the pancreas on day 0 and the chemotherapy started on day 4 **(A)**. The tumor (white box or arrow) could be identified by ^18^F-FDG-PET/CT on day 35 **(B,C)** and 7 T MRI on day 34 **(D)**. Tumors are surrounded by abundant collagen I deposition as indicated by double arrow **(E)**. Bar = 1 cm in **(C,D)**. Bar = 50 μm in **(E)**.

C57BL/6J male mice were purchased from The Jackson Laboratory (Bar Harbor, ME), and bread in our local animal facility. All processes of keeping mice and performing experiments were in accordance with the EU-directive 2010/63/EU, and approved by the local animal care committee (Landesamt für Landwirtschaft, Lebensmittelsicherheit und Fischerei Mecklenburg-Vorpommern). The syngeneic orthotopic pancreatic cancer model was performed as described previously (19). Briefly, after 1 week of accustoming to the environment, 2.5 × 10^5^ 6606PDA were injected into the pancreas of mice (Figure 1A). To relief pain 5 mg/kg carprofen (Pfizer GmbH, Berlin, Germany) was injected (sc) before surgery and 1,250 mg/L metamizol (Ratiopharm GmbH, Ulm, Germany) was added to the drinking water until euthanasia of the mice. On day 4 after cell injection, mice were daily treated (i.p.) with vehicle solution (Sham), CHC, 125 mg/kg metformin or CHC plus metformin. To evaluate an appropriate CHC dose for mice, three strategies, 15 mg/kg CHC plus 125 mg/kg metformin (*n* = 3), 60 mg/kg CHC plus 125 mg/kg metformin (*n* = 3), or 240 mg/kg CHC plus 125 mg/kg metformin (*n* = 3), were evaluated. In order to evaluate inhibition of autophagy *in vivo*, mice were i.p. injected with 60 mg/kg CQ or an appropriate volume of PBS twice per week. On day 37, after euthanasia, the tumor was separated from the pancreas and the weight was recorded.

### 7 T MRI and ^18^F-FDG-PET/CT Imaging

Anesthetized (1.2–2.5% isoflurane in oxygen) mice were scanned with a 7 T small animal MRI (BioSpec 70/30, 7.0 Tesla, gradient inset: BGA-12S, max. gradient strength: 440 mT/m) in combination with a transmit volume-resonator (86 mm inner diameter) and receive surface-coil (Bruker BioSpin GmbH, Ettlingen) on day 34 after 6606PDA cells injection (as indicated in Figure 1A). Animals were scanned using morphological T2 weighted TurboRARE (T2w-TurboRARE) and diffusion weighted imaging (DWI) sequences with following parameters: transversal T2w TurboRARE (Rapid Acquisition with Relaxation Enhancement): TE/TR: 25/1880 ms; FoV: approx. 40 × 28 mm; matrix: 200 × 200; voxel size: 0.2 × 0.14 mm, slice thickness 1 mm, 25 slices; transversal DWI-spin-echo sequence: 4 b values (*b* = 100, 300, 700, 1,000 s/mm^2^), one A0 image; 3 directions; TE/TR: 22/2500 ms; FoV: 28 × 20 mm, matrix: 128 × 128; voxel size: 0.22 mm × 0.156 mm, slice thickness 0.9 mm; 12 slices. On day 35 after 6606PDA cells injection, the ^18^F-FDG-PET/CT imaging was performed. Mice were anesthetized as mentioned above. Under anesthesia, the mice were injected with ~15 MBq of ^18^F-FDG intravenously via a microcatheter placed in a tail vein. After an uptake period of 60 min, static PET scans in head-prone position were acquired for 15 min using a small animal micro PET/CT scanner (Inveon PET/CT Siemens, Knoxville, TN, USA). Throughout the imaging session, respiration of the mice was controlled and body temperature was constantly kept at 38°C via a heating pad. The PET image reconstruction method consisted of a 2-dimensional ordered subset expectation maximization algorithm (2D-OSEM) with four iterations and 6 subsets. Attenuation correction was performed on the basis whole body CT scan and a decay correction for ^18^F was applied. PET images were also corrected for random coincidences, dead time and scatter.

### Immunohistochemical Staining

To evaluate the stromal reaction, collagen I staining was performed on 4 μm paraffin sections using rabbit anti-collagen I antibody (dilution: 200 ×) and alkaline phosphatase-conjugated goat anti-rabbit immunoglobulins (dilution: 100 ×), followed by permanent staining. All images of collagen I were obtained by an Olympus microscope, BX51, equipped with a ColorView II camera (Olympus, Tokyo, Japan). To measure the thickness of stromal reaction, the distance from the outside to the inside edge of collagen I deposition was measured at 12 o'clock, 3 o'clock, 6 o'clock and 9 o'clock position of the section. The mean value of these four distances was used to define the thickness of stromal reaction.

### Analysis of Blood

To evaluate the concentration of alanine aminotransferase (ALT), alanine transaminase (AST), and lipase in plasma, blood samples were taken before euthanasia of the mice and analyzed using the Cobas c111 analyzer (Roche Diagnostics, Mannheim, Germany).

### Statistics

The results were presented as box plots. The Mann-Whitney rank sum test, followed by Bonferroni correction determined the significance of differences. Differences with *P* ≤ 0.05, divided by the number of meaningful comparisons, were considered to be significant. All statistics were performed by Sigmaplot 12.0 (Systat Software, San Jose, CA, USA).

## Results

### An *in vivo* Model Replicating Features of Human Pancreatic Cancer

In order to evaluate if injection of 6606PDA cells gives rise to tumors with characteristic features of human pancreatic cancer, tumors were assessed by *in vivo* imaging and histological studies (Figure 1). We found that tumors could be identified by ^18^F-FDG-PET/CT (Figures 1B,C) and 7 T MRI (Figure 1D). To monitor the stromal reaction around the tumor, collagen I immunohistochemistry was performed. We observed that the carcinoma was surrounded by extensive collagen I deposition (Figure 1E).

### CHC Plus Metformin Inhibits Proliferation and Induces Cell Death

**Figure 2:**
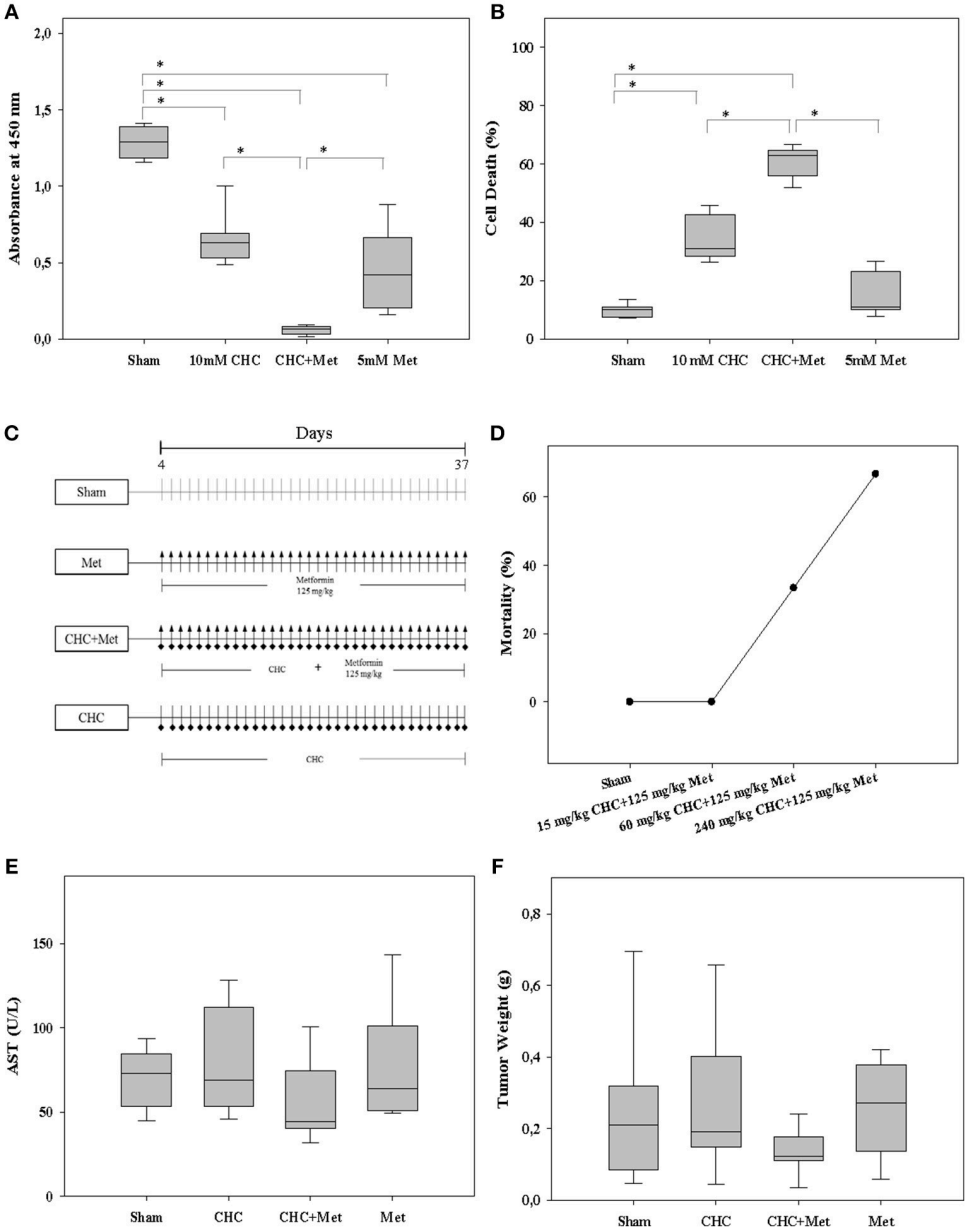
Efficacy and safety of drugs. The monotherapies, CHC or metformin (Met), and the combinatorial therapy using both drugs significantly inhibited cell proliferation *in vitro* **(A)**. These drugs also induced cell death **(B)**. Distinct drugs or the appropriate vehicle (Sham) was i.p. injected daily into mice **(C)**. 15 mg/kg CHC in combination with 125 mg/kg metformin was safe for mice **(D)**. These drugs did not significantly increase the AST activity, a liver toxicity indicator, in blood plasma **(E)**. CHC plus metformin slightly decreased the tumor weight **(F)**. *P ≤ 0.008.

In order to assess if inhibitors of cell metabolism impair pancreatic cancer cells, 6606PDA cells were treated with 10 mM CHC, 5 mM metformin or both drugs. We observed that the monotherapies significantly inhibited the proliferation of 6606PDA cells, compared to Sham-treated cells. The combination therapy, CHC plus metformin, also significantly inhibited proliferation, when compared to both monotherapies and Sham treatment (Figure 2A). In addition, we observed that 10 mM CHC significantly induced cell death while 5 mM metformin moderately increased the percentage of dead cells, when compared to cells treated with Sham (Figure 2B). Moreover, CHC in combination with metformin significantly induced cell death when compared to Sham, CHC, or metformin treated cells (Figure 2B).

### CHC Plus Metformin Is Safe for Mice and Slightly Impairs Pancreatic Cancer

**Figure 3:**
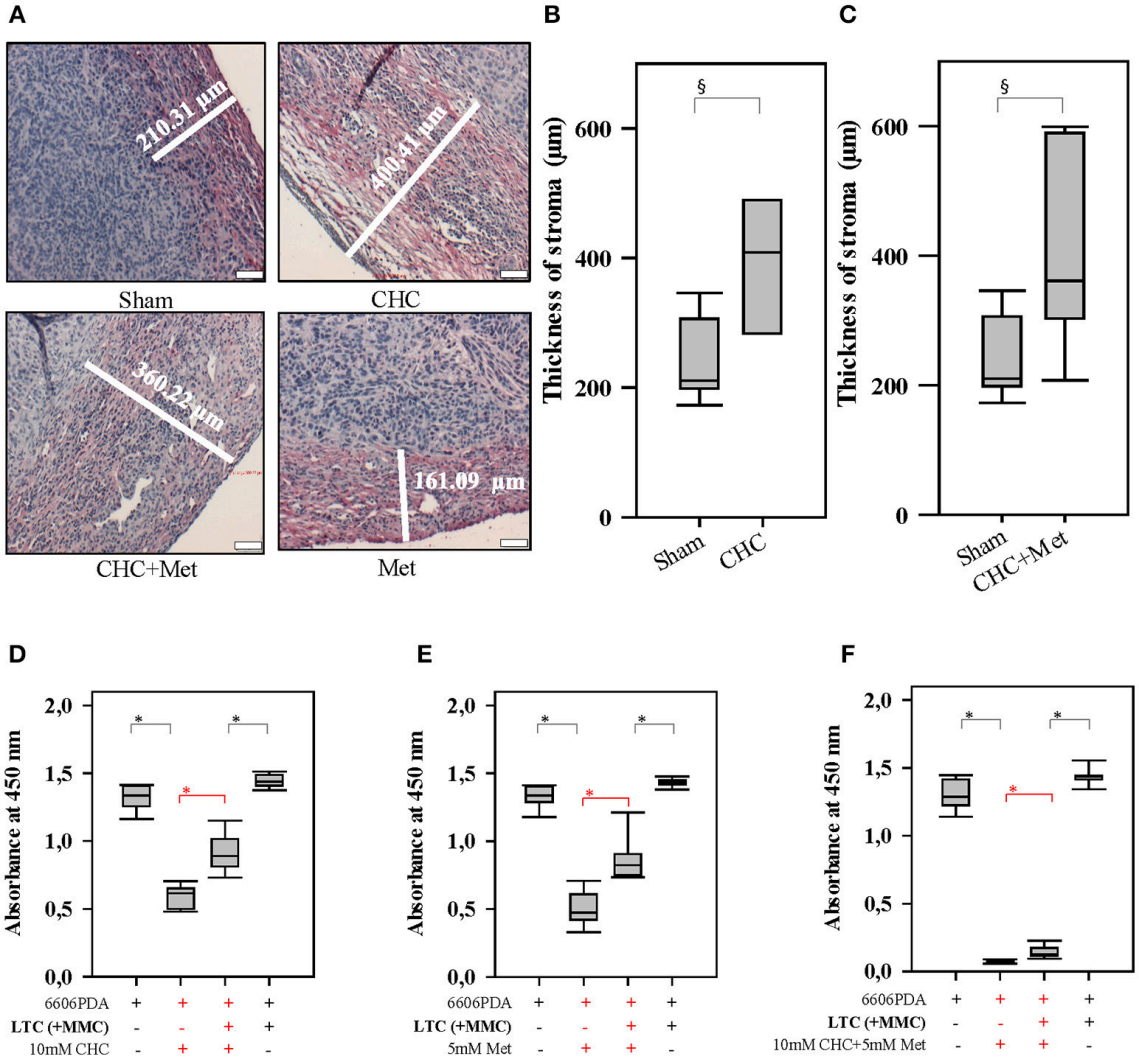
Drugs influence the tumor stroma and CAFs influence the sensitivity to drugs. CHC and CHC plus metformin (Met) significantly induced the stromal reaction (collagen I deposition stained in red) when compared to Sham treatment **(A–C)**. While CHC **(D)**, metformin **(E)**, or CHC plus metformin **(F)** treatment inhibited the proliferation of pancreatic cancer cells, the addition of LTC cells (their proliferation was inhibited by pretreatment with MMC) significantly (shown in red) reduced this anti-proliferative effect. ^§^*P* ≤ 0.05, *P ≤ 0.0125. Bar = 50 μm.

To determine an appropriate drug dose for mice, we evaluated three strategies, 15 mg/kg CHC plus 125 mg/kg metformin (low dose), 60 mg/kg CHC plus 125 mg/kg metformin (moderate dose) and 240 mg/kg CHC plus 125 mg/kg metformin (high dose) using the 6606PDA syngeneic orthotopic pancreatic cancer model (Figure 2C). We observed that only few mice survived after treating them with high dose and moderate dose of therapeutics (Figure 2D). However, all mice survived in the low dose group (Figure 2D). In addition, treatment with 15 mg/kg CHC, 125 mg/kg metformin, and the combination of both drugs did not significantly increase the AST and ALT activity, two indicators of liver toxicity in blood plasma (Figure 2E and Figure S1A). These drugs also failed to significantly increase lipase activity, an indicator of inflammation in the pancreas (Figure S1B). Thus, we chose 15 mg/kg CHC to evaluate the benefit of CHC and metformin *in vivo*. We observed a minor decrease in tumor weight after treating mice with CHC or CHC plus metformin when compared to Sham-treated mice (Figure 2F). Interestingly, we also observed that CHC or CHC plus metformin therapy significantly increased the thickness of stroma as defined by collagen I deposition (Figures 3A–C). However, there was no significant difference between metformin treated and Sham-treated tumors. This suggest that CHC activates CAFs to produce more collagen. This observation triggered speculations that these CAFs can impair the anti-proliferation efficacy of CHC and metformin.

### Inhibition of Autophagy in CAFs Enhances the Efficacy of Chemotherapeutical Agents

**Figure 4:**
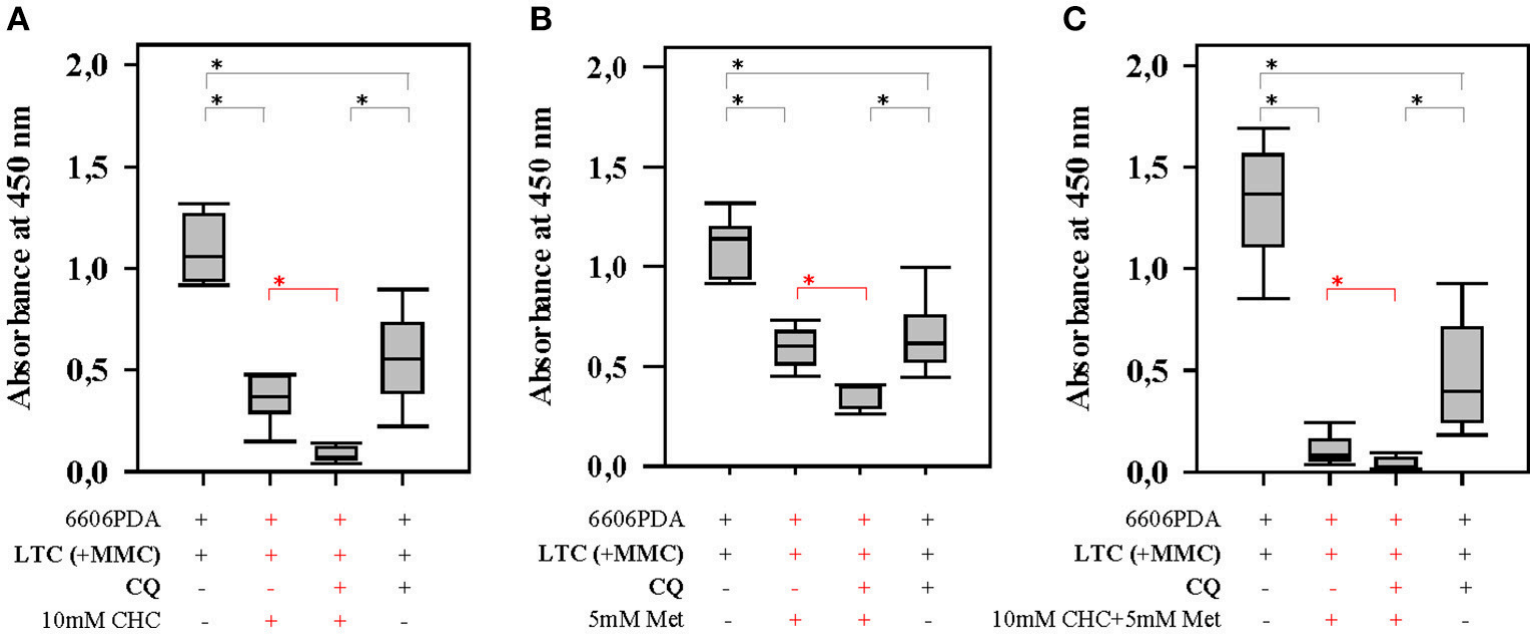
Autophagy in CAFs attenuates the anti-proliferation activity of CHC and metformin (Met). Blocking autophagy in LTC cells (their proliferation was inhibited by pretreatment with MMC) with CQ significantly (shown in red) increased the sensitivity of pancreatic cancer cells to CHC **(A)**, metformin **(B)**, or CHC plus metformin **(C)**. *P ≤ 0.0125.

In order to address the question, if CAFs are capable of impairing the anti-proliferation effect of chemotherapeutical agents, we pursued *in vitro* experiments. We co-cultured LTC cells and 6606PDA cells. Subsequently, these co-cultured cells were treated with CHC, metformin or a combinatorial treatment. We observed that LTC cells impaired the anti-proliferative effect of CHC, metformin, and CHC plus metformin without having a major influence on the proliferation of untreated cancer cells (significant difference is shown in red in Figures 3D–F). This suggests that LTC cells induce chemoresistance in pancreatic cancer cells. In order to evaluate if autophagy in CAFs contributes to the observed chemoresistance, we pretreated LTC cells with 50 μM CQ, a well-known inhibitor of autophagy. Interestingly, after blocking autophagy in LTC cells, the co-cultured pancreatic cancer cells were more sensitive to CHC, metformin, and CHC plus metformin (significant difference is shown in red in Figure 4).

**Figure 5:**
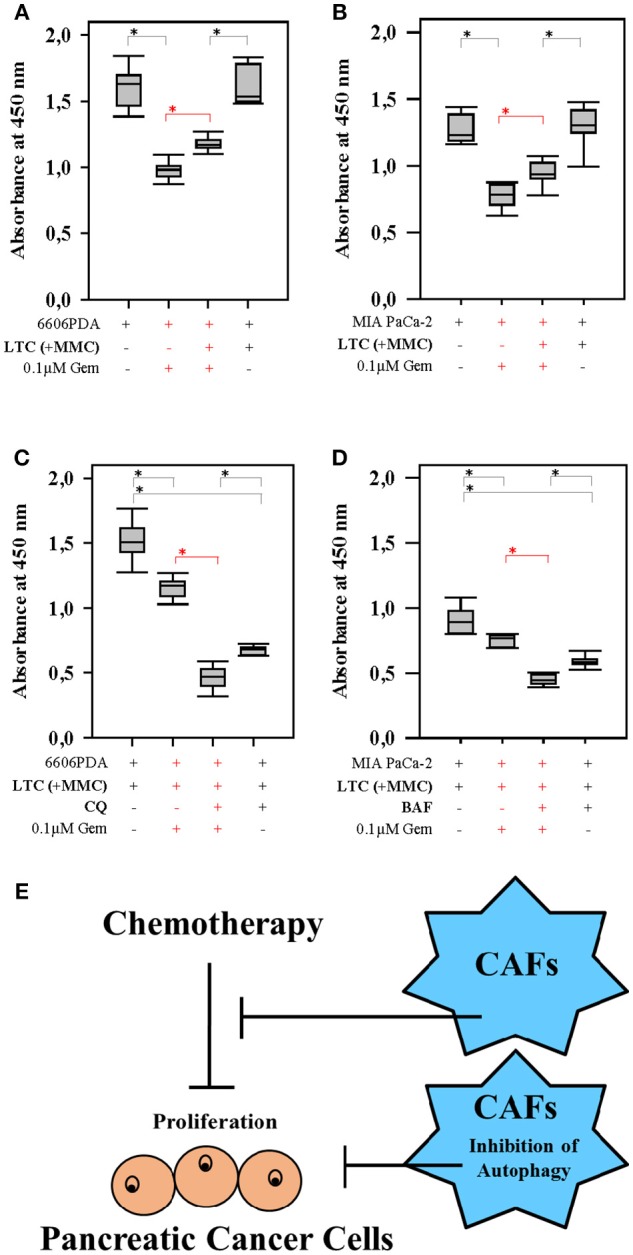
Autophagy in CAFs inhibits the anti-proliferative effect of gemcitabine (Gem). Co-culture with LTC cells (their proliferation was inhibited by pretreatment with MMC) significantly (shown in red) reduced the anti-proliferative effect of gemcitabine on 6606PDA cells **(A)** and MIA PaCa-2 cells **(B)**. The pancreatic cancer cells were significantly (shown in red) more sensitive to gemcitabine, after inhibiting autophagy in LTC cells by CQ **(C)**, or BAF **(D)**. The present study demonstrates two points. First, CAFs impair the efficacy of chemotherapies. Second, blocking autophagy in CAFs supports the anti-proliferation activity of chemotherapeutic drugs **(E)**. *P ≤ 0.0125.

Next we verified, if inhibition of autophagy in CAFs also improved the efficacy of gemcitabine, a first-line therapy for the treatment of pancreatic cancer. When pancreatic cancer cells were co-cultured with LTC cells, we observed that LTC cells significantly reduced the anti-proliferative effect of gemcitabine in two distinct cell line, 6606PDA and MIA PaCa-2 (significant difference is shown in red in Figures 5A,B). However, LTC cells did not have a major influence on the proliferation of untreated cancer cells (Figures 5A,B). This suggests that LTC cells induce resistance to gemcitabine in pancreatic cancer cells. After blocking autophagy in LTC cells with CQ, the co-cultured pancreatic cancer cells were more sensitive to gemcitabine (significant difference is shown in red in Figure 5C). We confirmed this result by using another inhibitor of autophagy, BAF. Indeed, after blocking autophagy in LTC cells with BAF, the co-cultured pancreatic cancer cells were also more sensitive to gemcitabine (significant difference is shown in red in Figure 5D). These data suggests that we observe two distinct processes (Figure 5E). First, CAFs do not significantly stimulate the proliferation of carcinoma cells, but protect carcinoma cells form chemotherapeutical agents. A separate process is the inhibition of autophagy. Blockage of autophagy in CAFs inhibits cancer cell proliferation.

**Figure 6:**
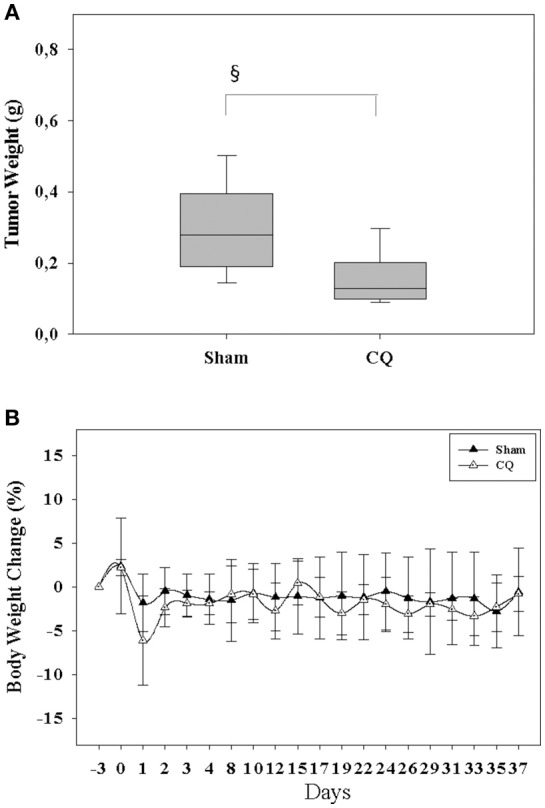
Inhibition of autophagy reduces tumor weight and is safe. Treating mice with CQ reduced the tumor weight **(A)** without having a major influence on body weight **(B)**. ^§^*P* ≤ 0.05.

We next assessed in an *in vivo* proof of principle experiment, if inhibition of autophagy is well-tolerated and can reduce tumor growth. We injected 6606PDA cells into the pancreas of mice and treated one cohort of mice with CQ. Therapy with CQ reduced the tumor weight significantly compared to Sham treated mice (Figure 6A), without causing any obvious burden to mice as demonstrated by little change in body weight throughout the experiment (Figure 6B).

### Chemotherapeutical Agents Modulate Autophagy

**Figure 7:**
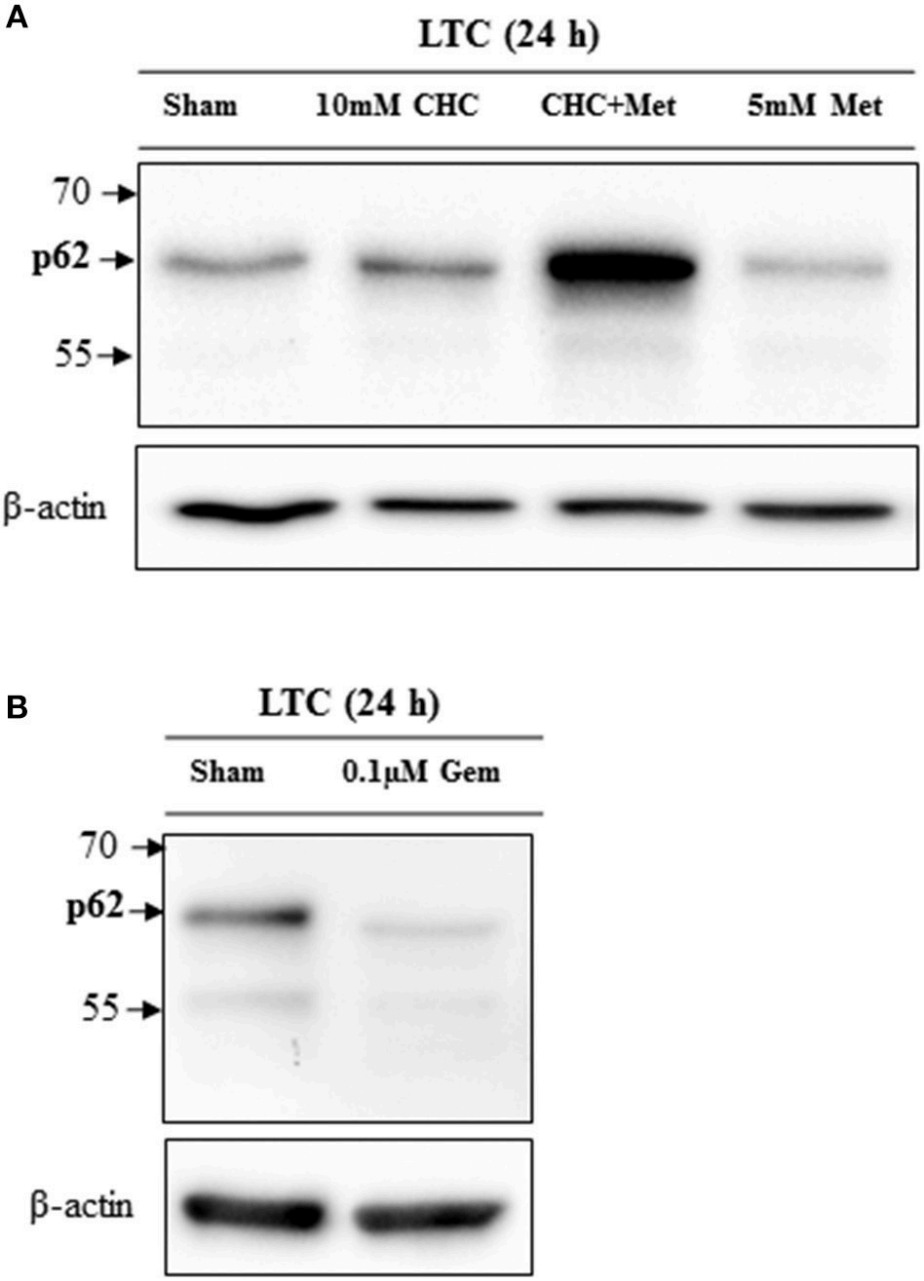
Drugs influence the autophagic flux in LTC cells. CHC and CHC plus metfomin (Met) treatment caused accumulation of p62 whereas metformin treatment reduced p62 level **(A)**. Gemcitabine (Gem) treatment reduced the p62 level **(B)**.

In order to assess, if chemotherapies have an influence on autophagy, we treated LTC cells with metformin, CHC, the combinational therapy or gemcitabine. Both, CHC and CHC plus metformin treatment increased the accumulation of p62 (Figure 7A). This suggests that these chemotherapies block the autophagy flux. However, we observed that metformin decreased the level of p62 (Figure 7A) and, therefore, induced the autophagy flux. In addition, we observed that gemcitabine decreased the accumulation of p62 in LTC cells (Figure 7B), which suggests that gemcitabine induces autophagy in these cells. This proposes that distinct agents can have opposite effects on autophagy in CAFs.

## Discussion

The present study demonstrates that CAFs protect carcinoma cells form chemotherapeutical agents. It is well-known that pancreatic cancer is usually surrounded by an extensive stromal reaction containing CAFs and that these CAFs can influence the sensitivity of carcinoma cells to chemotherapy (2, 20, 21). Originally, it was assumed that the stromal reaction forms a physical barrier to protect cancer cells from chemotherapeutics (22–25). Indeed, we also noticed, in our orthotopic cancer model, an intense barrier like stromal reaction (Figure 3A) and only moderate reduction of tumor weight in response to chemotherapies (Figure 2F). We observed that LTC cells inhibit the anti-proliferation activity of chemotherapeutic drugs *in vitro* (Figures 3D–F, 5A,B). This argues for an active function of CAFs in controlling chemoresistance of cancer cells.

In addition, the present study demonstrates that the blockage of autophagy in CAFs inhibits cancer cell proliferation. We observed this inhibition of proliferation in the presence, but also in the absence of drugs such as CHC, metformin and gemcitabine (Figures 4, 5C,D). This suggests that this effect is very robust. Indeed, our data demonstrate that this anti-proliferative effect is also, for example, independent of the effect of chemotherapeutic drugs on autophagy. We and other studies suggest that metformin and gemcitabine induce autophagy (26, 27). Surprisingly, we also observed that other treatments such as CHC and CHC in combination with metformin block autophagic flux in CAFs. Nevertheless, our data demonstrate that blocking autophagic flux in CAFs inhibits proliferation of cancer cells, independent if these chemotherapeutic agents induce or inhibit autophagy. Possibly, the synergistic anti-proliferation effect of CQ and CHC treatment is caused by an additive effect of both drugs on the inhibition of autophagy. On the contrary, CQ enhances the anti-proliferation effect of metformin or gemcitabine by blocking their induction of autophagy. Moreover, we noticed that CQ can also inhibit directly cancer cell proliferation, even when cancer cells are not co-cultured with CAFs (data not shown). This suggests that the blockage of autophagy in both CAFs as well as cancer cells has the identical effect: The reduction of cancer cell proliferation. Both effects might reduce tumor size *in vivo* as demonstrated in Figure 6A.

Many preclinical studies demonstrated that the strategy to abolish stromal reaction could increase the sensitivity of cancer cells to several chemotherapeutics (4, 7, 28). Unfortunately, some of these strategies, such as using vismodegib (GDC-0449), failed to give rise to a survival benefit for pancreatic cancer patients in clinical trials (8). In addition, Özdemir et al. demonstrated that depletion of CAFs results in multiple adverse outcomes and leads to a poor prognosis *in vivo* (29). This suggests that blocking or reducing the stromal reaction to cancer might not be part of a successful anti-cancer therapy. We suggest that modifying specific interactions between CAFs and cancer cells, for example by inhibition of autophagy in CAFs, may be a more promising strategy to treat pancreatic cancer. The following two therapeutical options could be explored in additional studies: First, one could evaluate if there are beneficial effects when inhibiting autophagy in CAFs in addition to manipulating other mechanisms, which have also been demonstrated to regulate cancer cell proliferation, survival or drug resistance. A promising strategy could be to regulate the SDF1 alpha/CXCR4 axis or mTOR/4E-BP1 signaling in CAFs (6, 30, 31). Second, one could evaluate in which cancer types inhibition of autophagy in combination with the first-line therapeutics has a beneficial effect.

Blocking autophagy in fibroblast like cells might also have therapeutic potential in other diseases. Recent studies suggest that increased autophagy contributes to various diseases, such as liver fibrosis or rheumatoid arthritis (32, 33). However, the function of autophagy in these diseases is still highly controversial (34). Thus, additional studies will be necessary to understand the contributions of autophagy in fibroblast-rich diseases, in order to provide a basis for novel therapies.

In conclusion, the present study demonstrated that blocking autophagy in CAFs successfully supports the anti-proliferation activity of three distinct drugs, CHC, metformin and gemcitabine, in pancreatic cancer cells.

## Author Contributions

DZ, BV, BK, and RJ: study concepts. XZ, DZ, and MS: study design; XZ, MS, DZ, JE, EW, SK, JS, and TL: data acquisition; XZ and DZ: quality control of data and algorithms; XZ and DZ: data analysis and interpretation; XZ: statistical analysis; XZ and DZ: manuscript preparation. All authors reviewed and edited the manuscript.

### Conflict of Interest Statement

The authors declare that the research was conducted in the absence of any commercial or financial relationships that could be construed as a potential conflict of interest.

## Supplementary Material

Figure S1 Drugs do not cause liver toxicity or inflammation in the pancreas. The monotherapies, CHC or metformin (Met), and the combinatorial therapy had only little influence on ALT activity **(A)** or lipase activity **(B)** in blood plasma of mice.

## Abbreviations

CAFs: cancer-associated fibroblasts
CHC: α-cyano-4-hydroxycinnamate
MMC: mitomycin C
CQ: chloroquine
BAF: bafilomycin A1.
