# Supplementary figures and images for: Blocking Autophagy in Cancer-Associated Fibroblasts Supports Chemotherapy of Pancreatic Cancer Cells
Source: Front Oncol. 2018 Dec 5;8:590. doi: 10.3389/fonc.2018.00590 (PMC6290725; doi:10.3389/fonc.2018.00590)

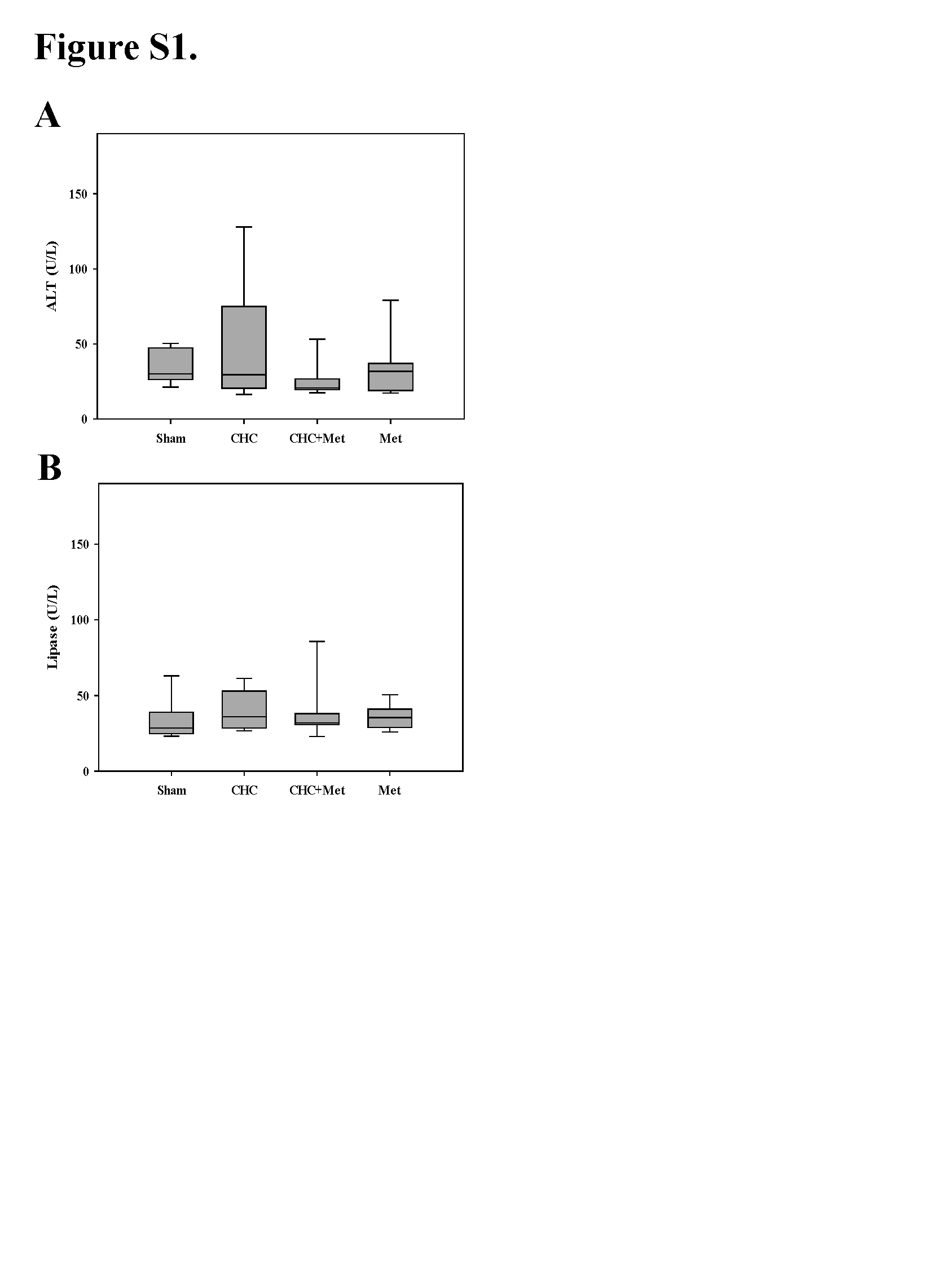

Supplement: Figure S1 — Drugs do not cause liver toxicity or inflammation in the pancreas. The monotherapies, CHC or metformin (Met), and the combinatorial therapy had only little influence on ALT activity (A) or lipase activity (B) in blood plasma of mice. [file Image_1.tiff]
